# Supplementary figures and images for: Structural and dynamic changes in P-Rex1 upon activation by PIP3 and inhibition by IP4
Source: eLife. 2024 Jul 31;12:RP92822. doi: 10.7554/eLife.92822 (PMC11290822; doi:10.7554/eLife.92822)

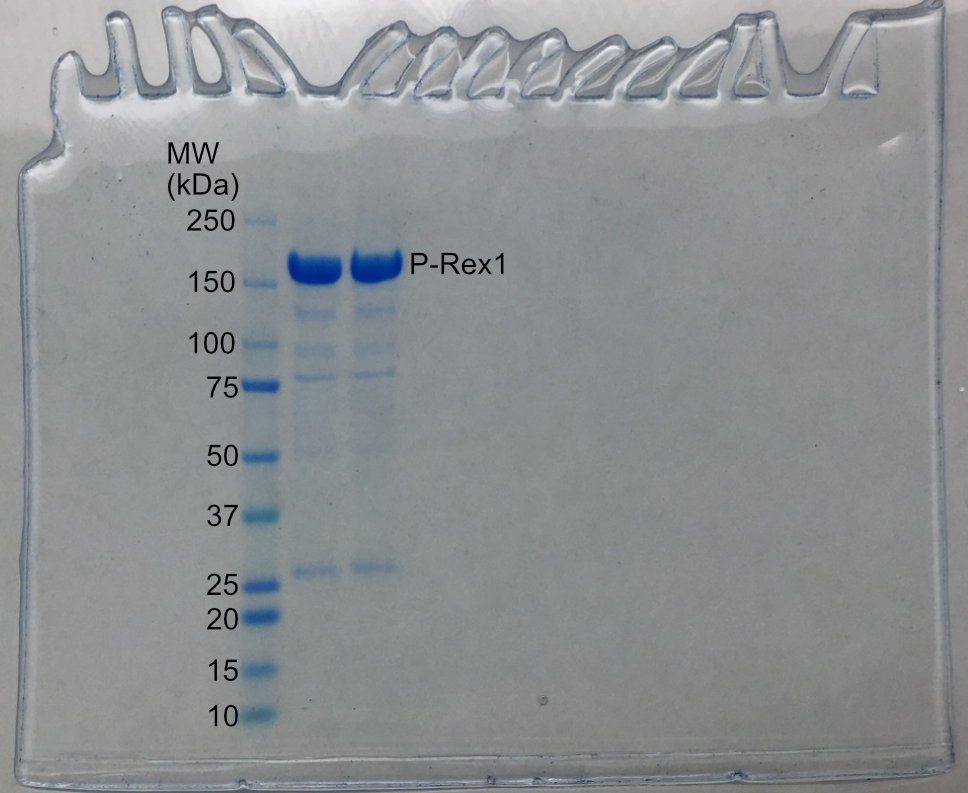

Supplement: Figure 2—figure supplement 1—source data 1. [file elife-92822-fig2-figsupp1-data1.zip › Figure2_FigureSupplement1ΓÇôSourceData2.tiff]

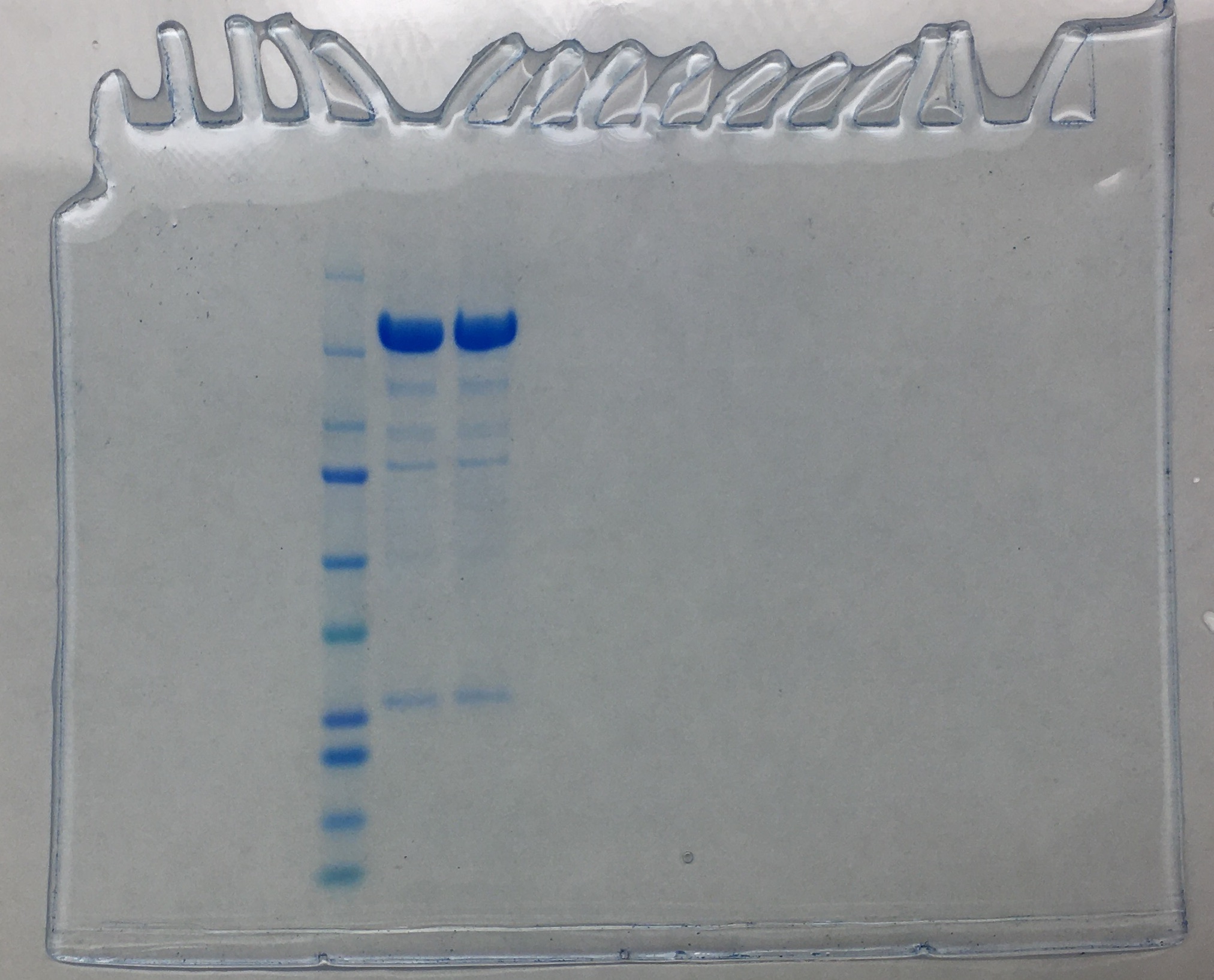

Supplement: Figure 2—figure supplement 1—source data 1. [file elife-92822-fig2-figsupp1-data1.zip › Figure2_FigureSupplement1ΓÇôSourceData1.JPG]

## Corresponding to Supplemental Figure 7C

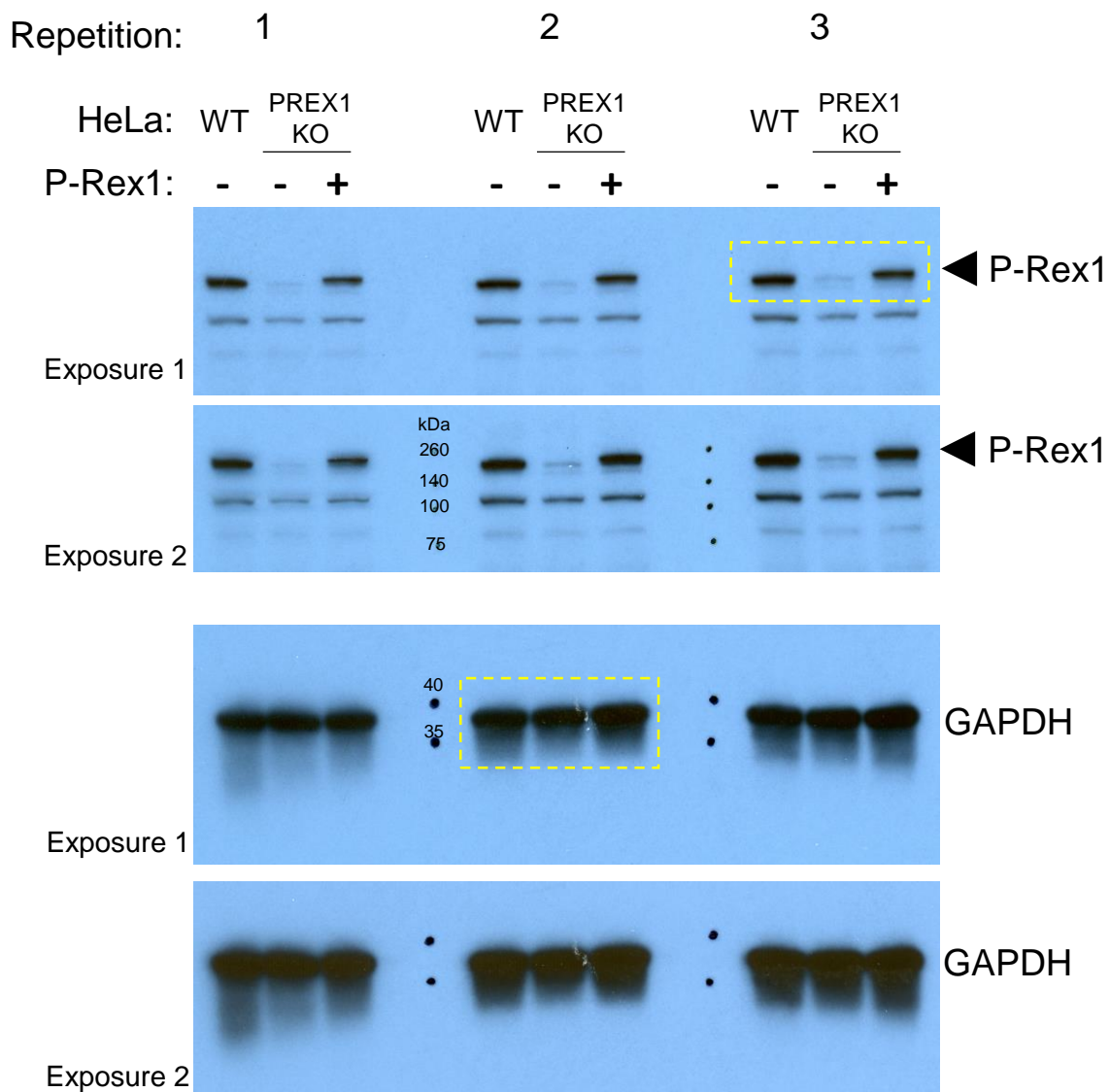

# Corresponding to Supplemental Figure 7D

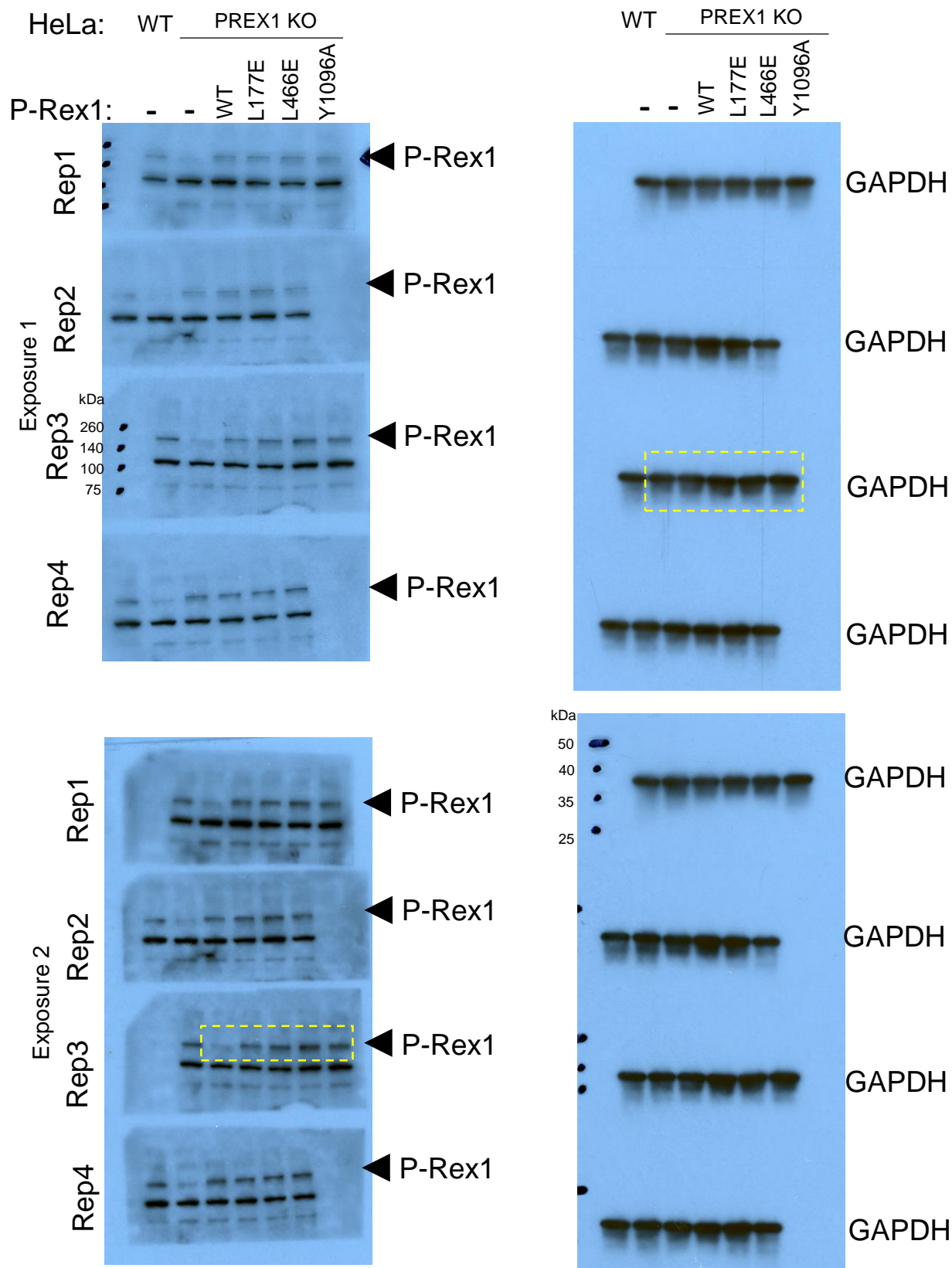

Supplement: Figure 5—figure supplement 1—source data 1. [file elife-92822-fig5-figsupp1-data1.zip › Figure5_FigureSupplement1-SourceData/Fig5_FigSup1_SourceData.pdf]

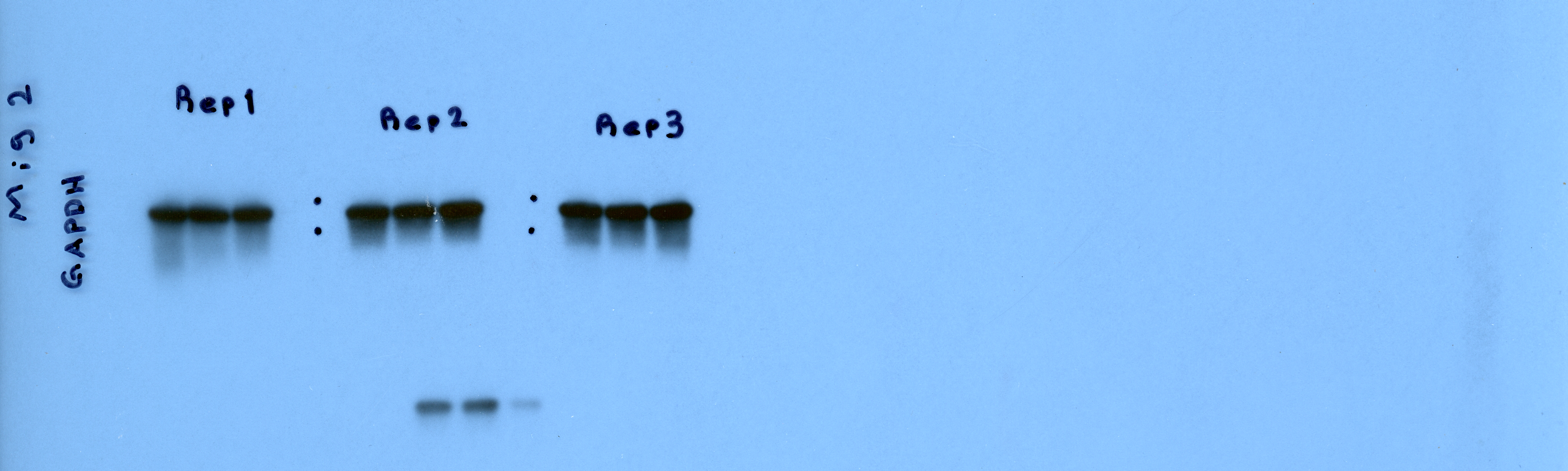

Supplement: Figure 5—figure supplement 1—source data 1. [file elife-92822-fig5-figsupp1-data1.zip › Figure5_FigureSupplement1-SourceData/PREX1 HeLa CRISPR KO (Rep 1-3)002.tif]

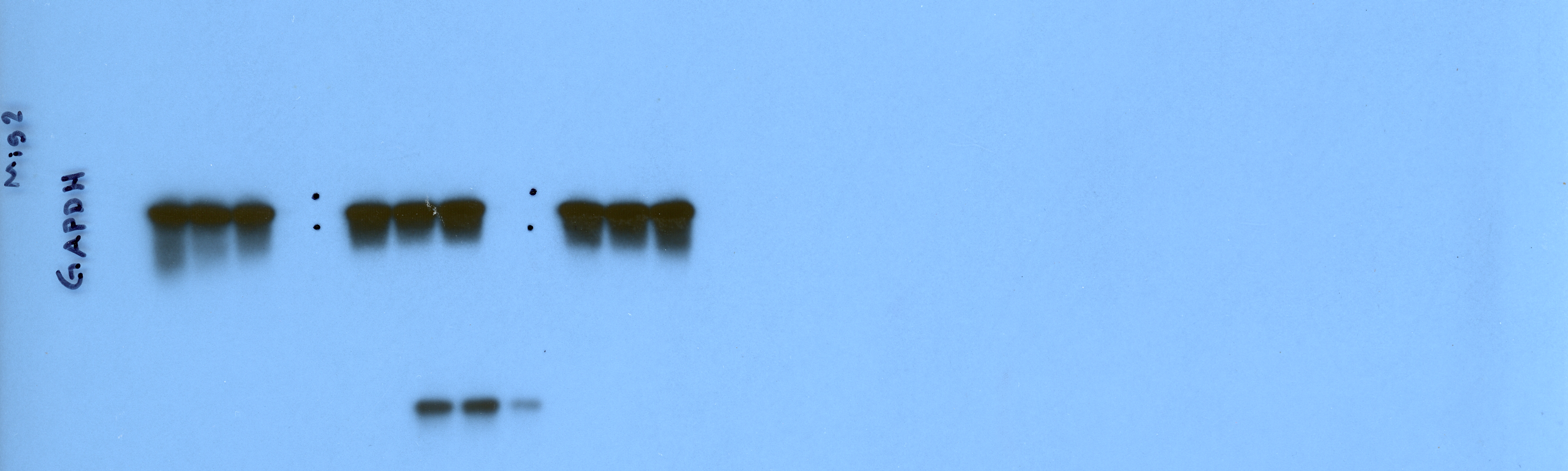

Supplement: Figure 5—figure supplement 1—source data 1. [file elife-92822-fig5-figsupp1-data1.zip › Figure5_FigureSupplement1-SourceData/PREX1 HeLa CRISPR KO (Rep 1-3)003.tif]

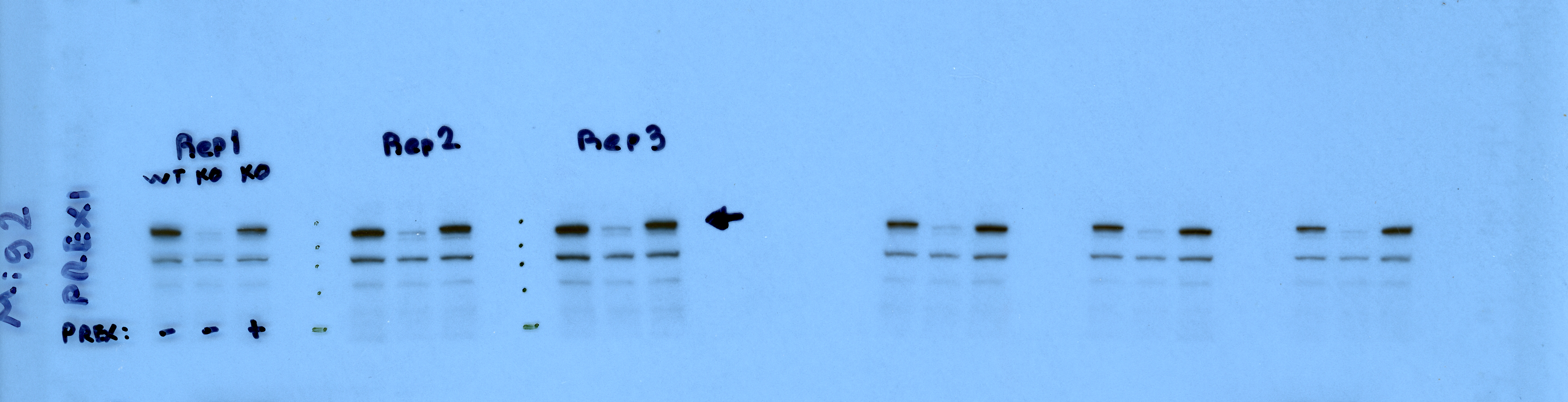

Supplement: Figure 5—figure supplement 1—source data 1. [file elife-92822-fig5-figsupp1-data1.zip › Figure5_FigureSupplement1-SourceData/PREX1 HeLa CRISPR KO (Rep 1-3)001.tif]

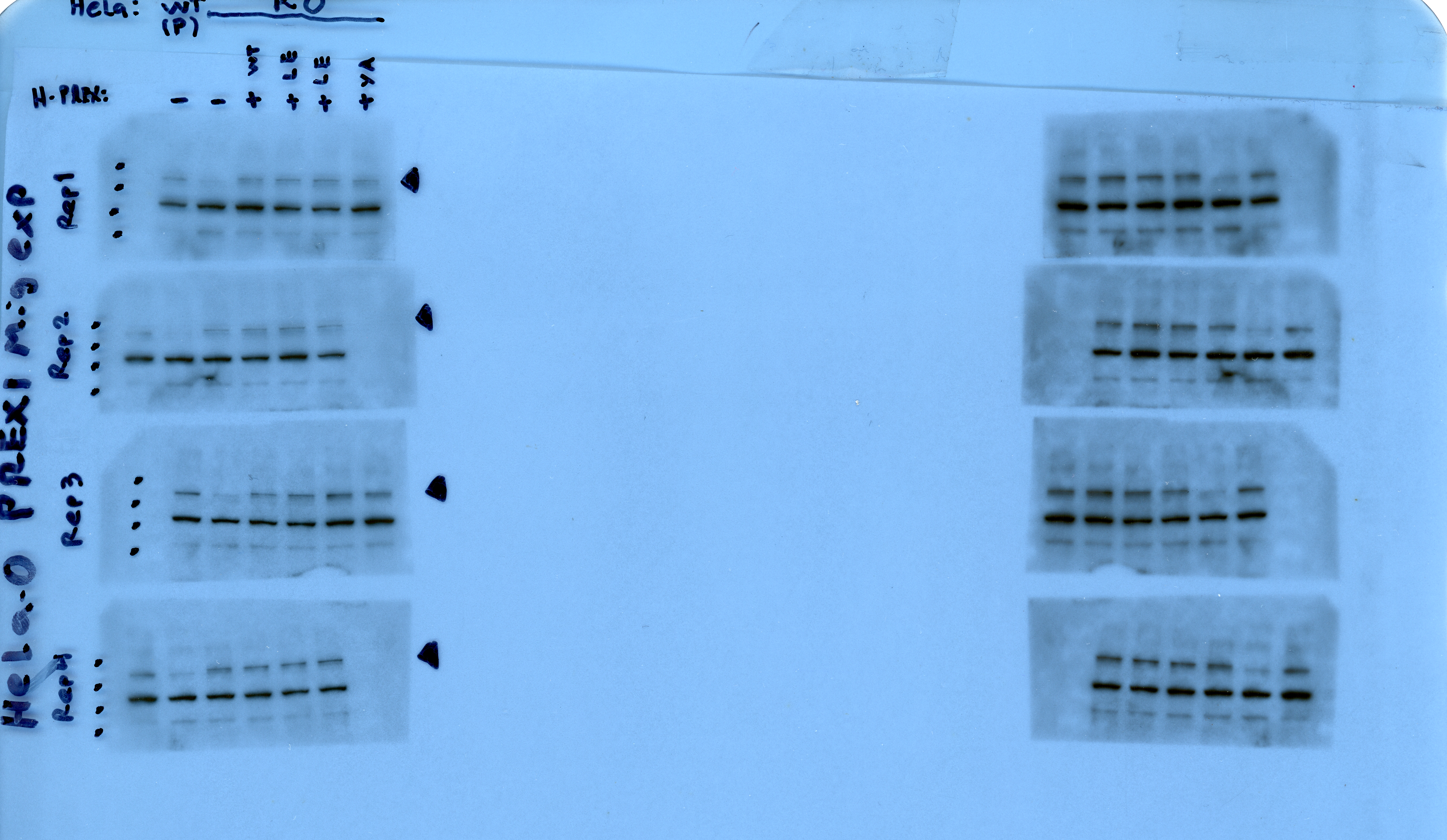

Supplement: Figure 5—figure supplement 1—source data 1. [file elife-92822-fig5-figsupp1-data1.zip › Figure5_FigureSupplement1-SourceData/PREX1 HeLa CRISPR KO mig ctrls (Rep 1-4)004.tif]

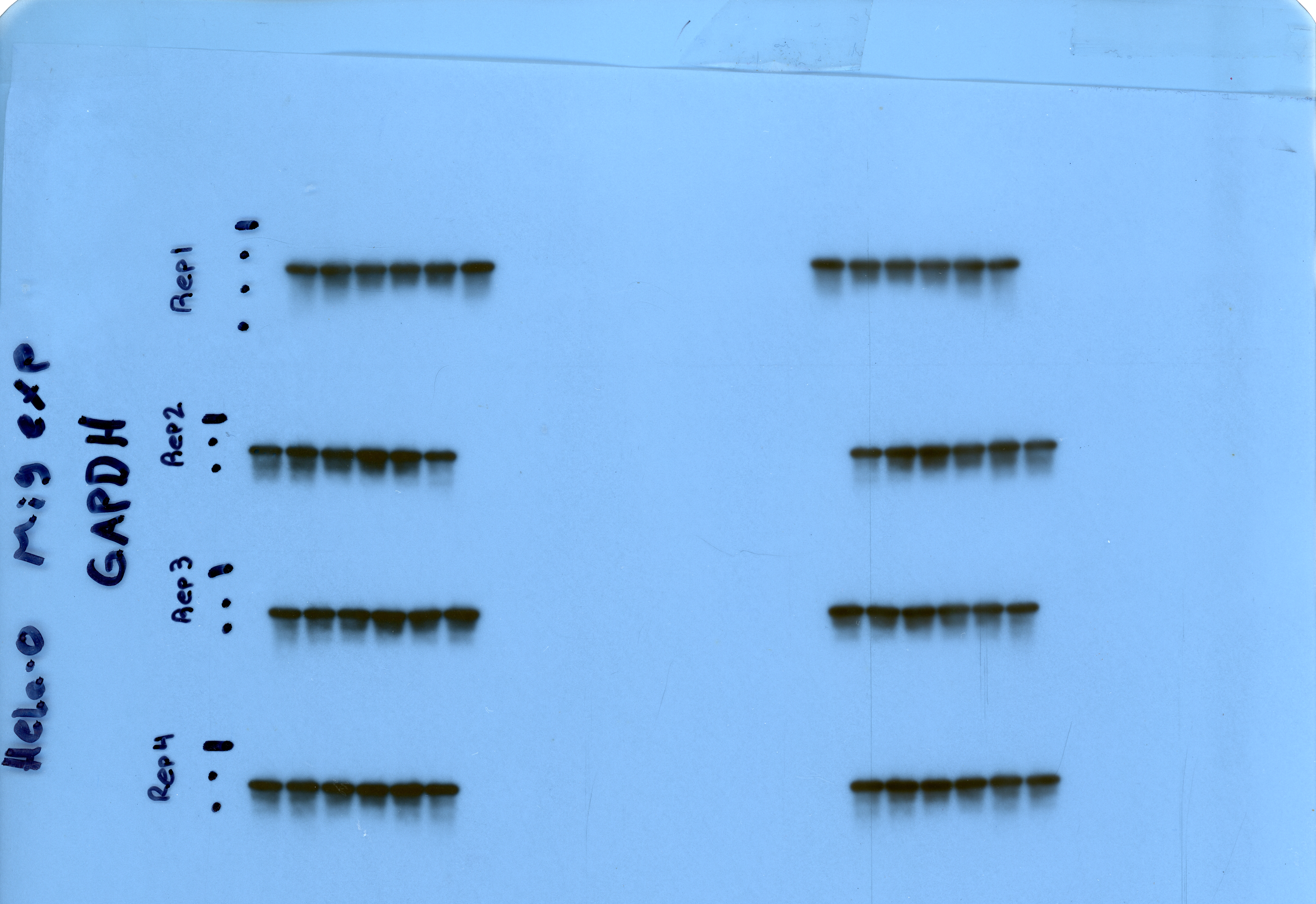

Supplement: Figure 5—figure supplement 1—source data 1. [file elife-92822-fig5-figsupp1-data1.zip › Figure5_FigureSupplement1-SourceData/PREX1 HeLa CRISPR KO mig ctrls (Rep 1-4)005.tif]

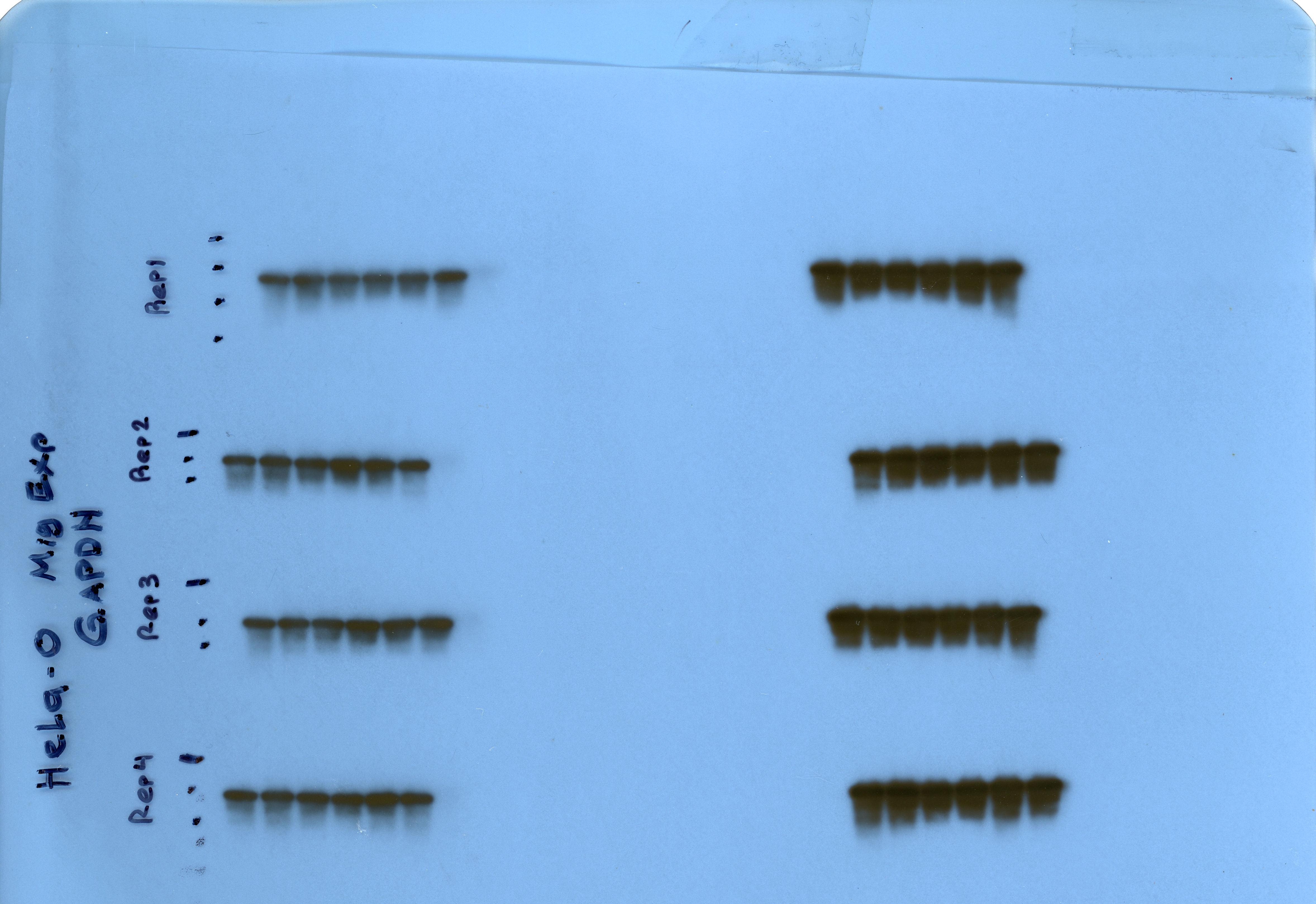

Supplement: Figure 5—figure supplement 1—source data 1. [file elife-92822-fig5-figsupp1-data1.zip › Figure5_FigureSupplement1-SourceData/PREX1 HeLa CRISPR KO mig ctrls (Rep 1-4)006.tif]
