# Supplementary material for: Structural and dynamic changes in P-Rex1 upon activation by PIP3 and inhibition by IP4
Source: eLife. 2024 Jul 31;12:RP92822. doi: 10.7554/eLife.92822 (PMC11290822; doi:10.7554/eLife.92822)
Supplement: Figure 4—source data 1. [file elife-92822-fig4-data1.pdf]

Ribbon Map of P-Rex1 (% deuteration)

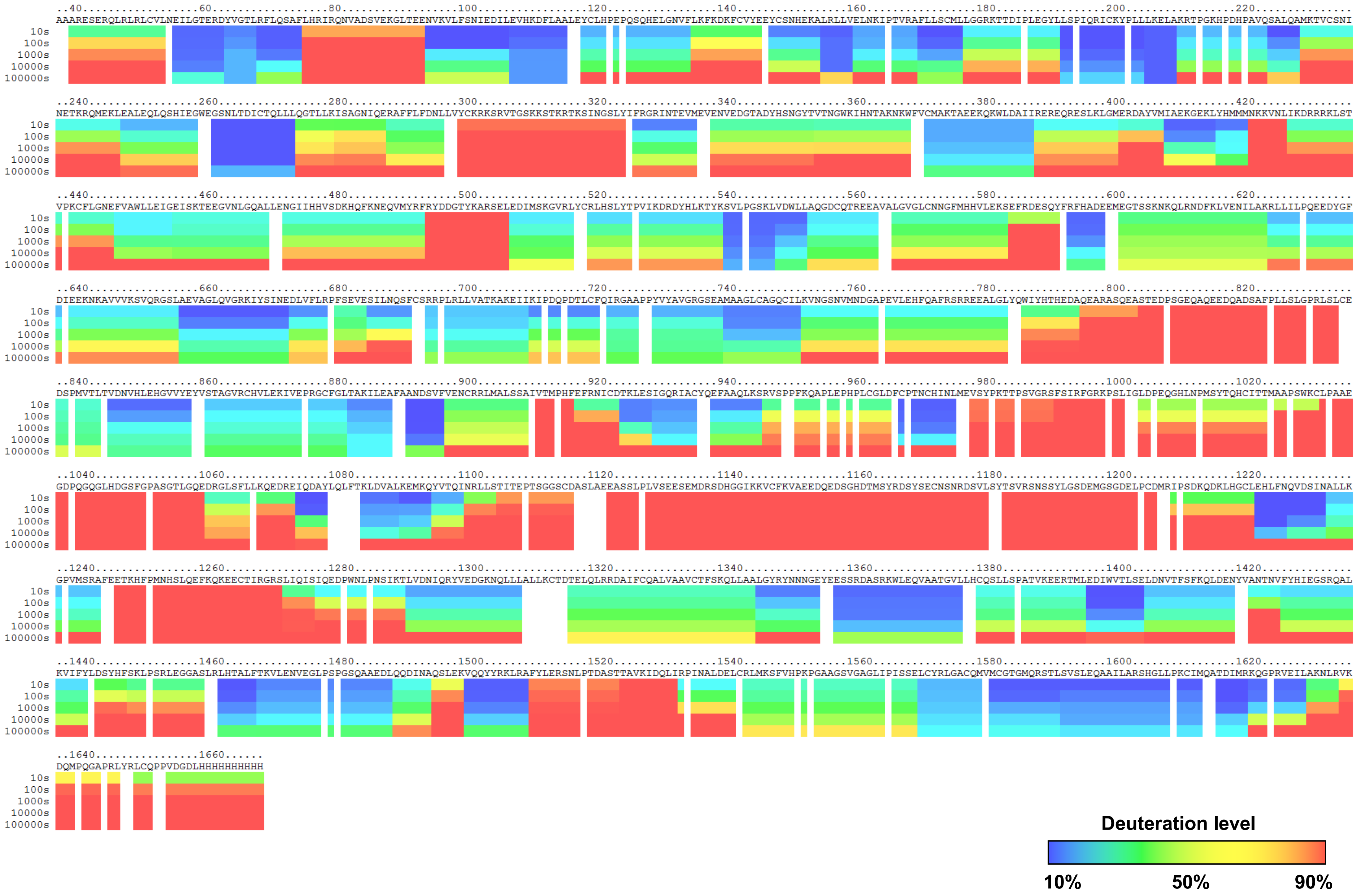

Ribbon Map of P-Rex1 in P-Rex1•IP$_4$ Complex (% deuteration)

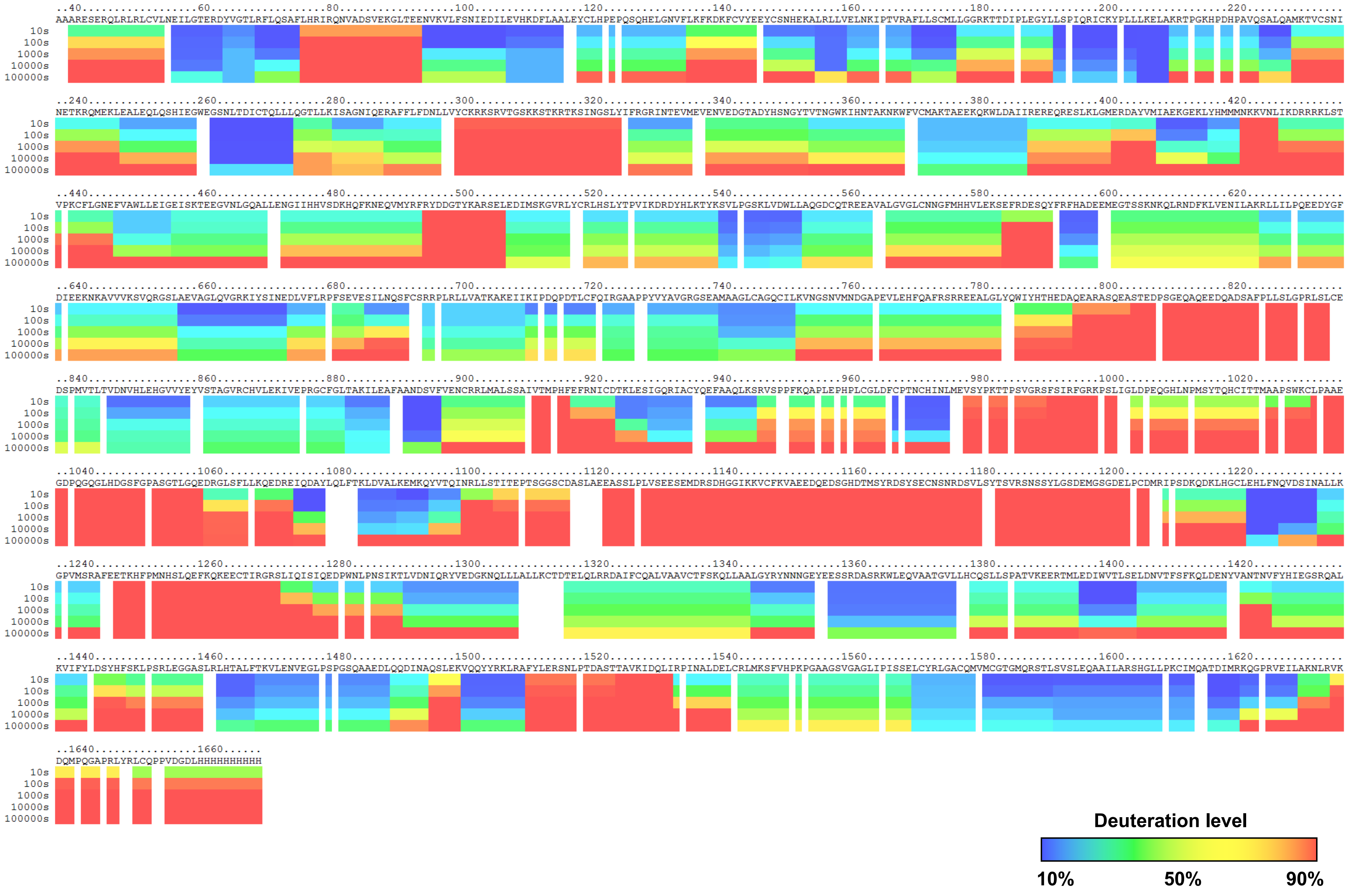

Influence of IP_4 on Exchange in P-Rex1 (% deuteration)

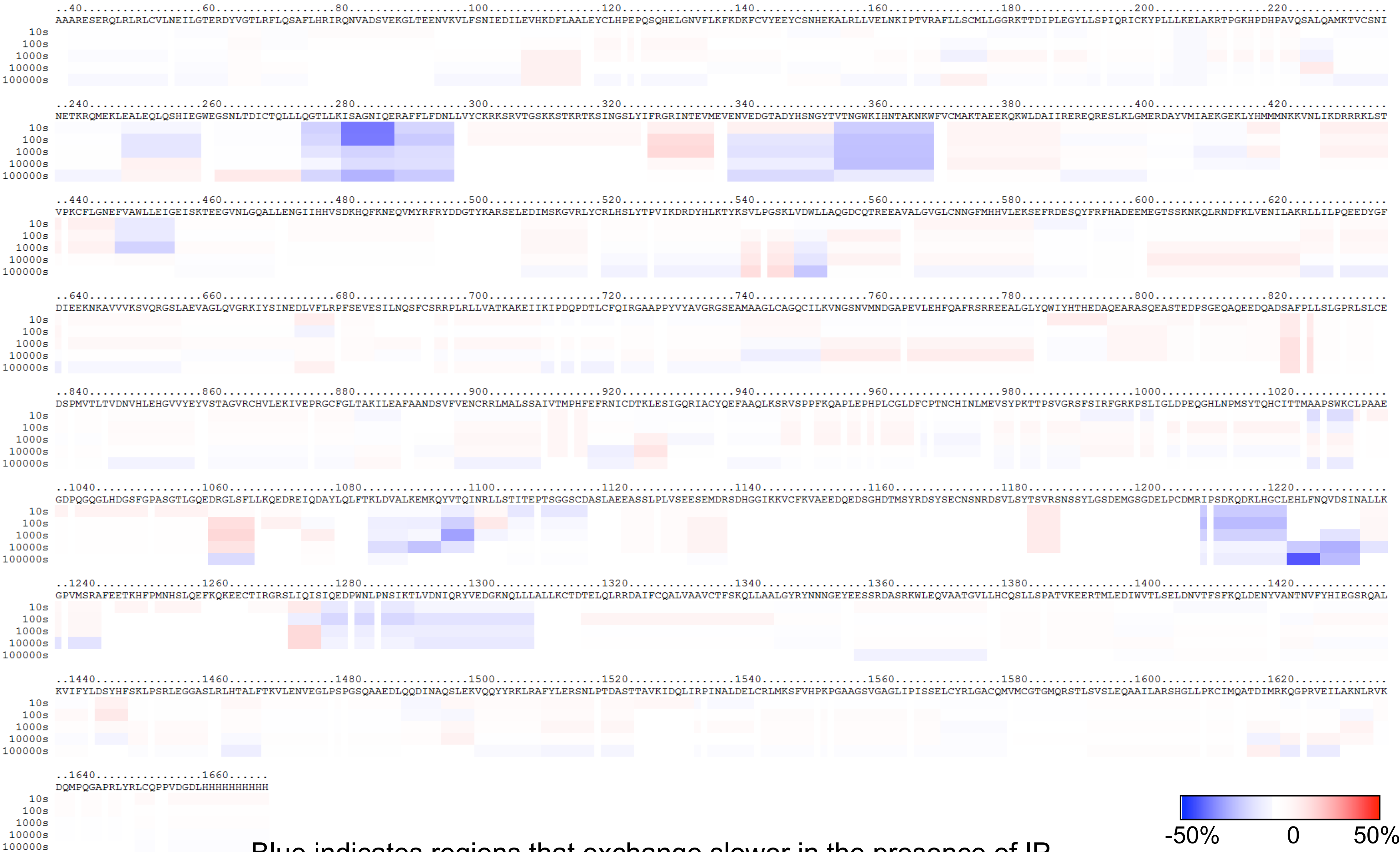

Blue indicates regions that exchange slower in the presence of IP_4.
Red indicates regions that exchange faster.
