# Supplementary material for: Structural and dynamic changes in P-Rex1 upon activation by PIP3 and inhibition by IP4
Source: eLife. 2024 Jul 31;12:RP92822. doi: 10.7554/eLife.92822 (PMC11290822; doi:10.7554/eLife.92822)

Ribbon Map of P-Rex1 (% deuteration)

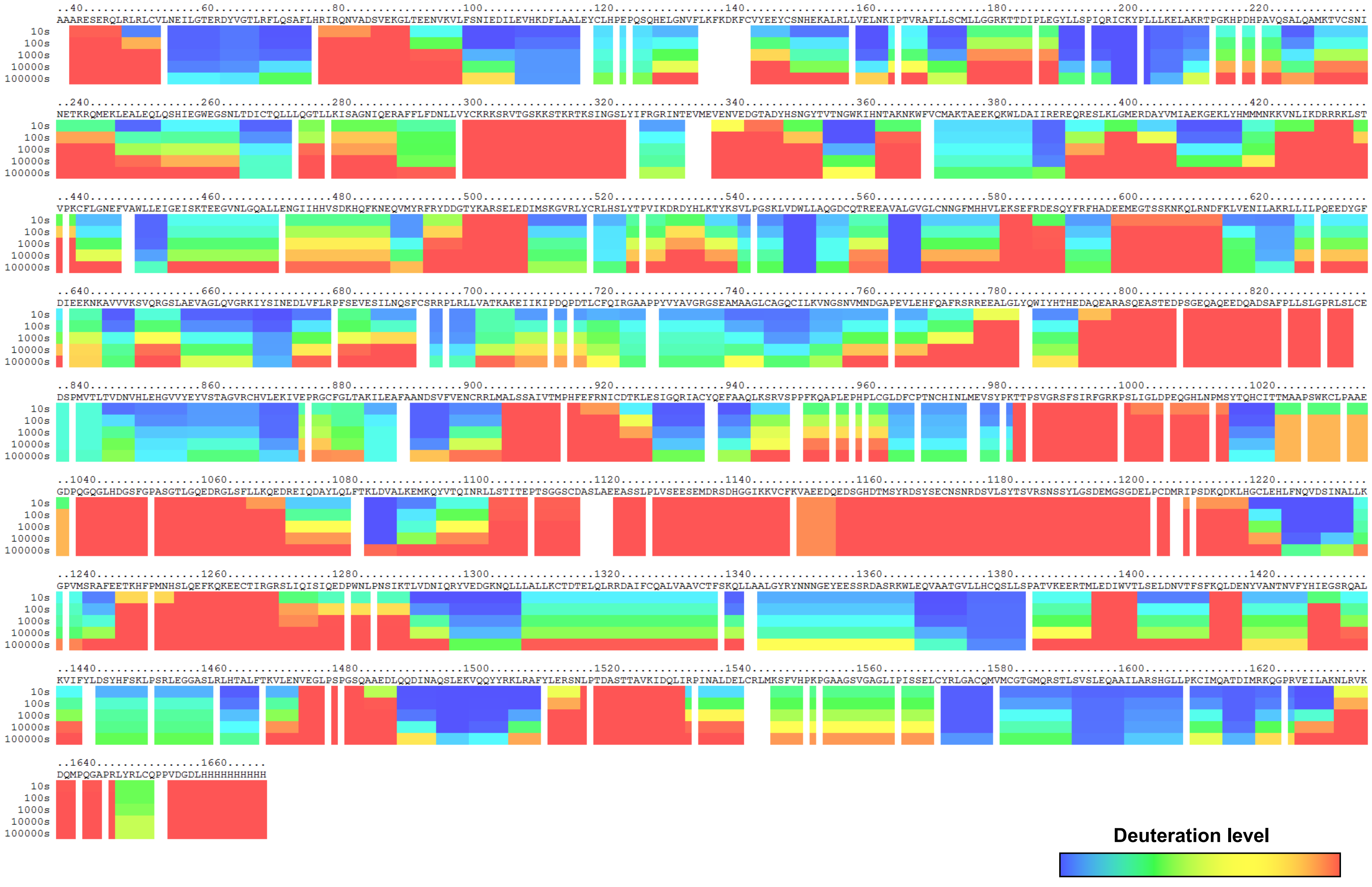

Ribbon Map of P-Rex1 in the presence of PIP_3-containing liposomes (% deuteration)

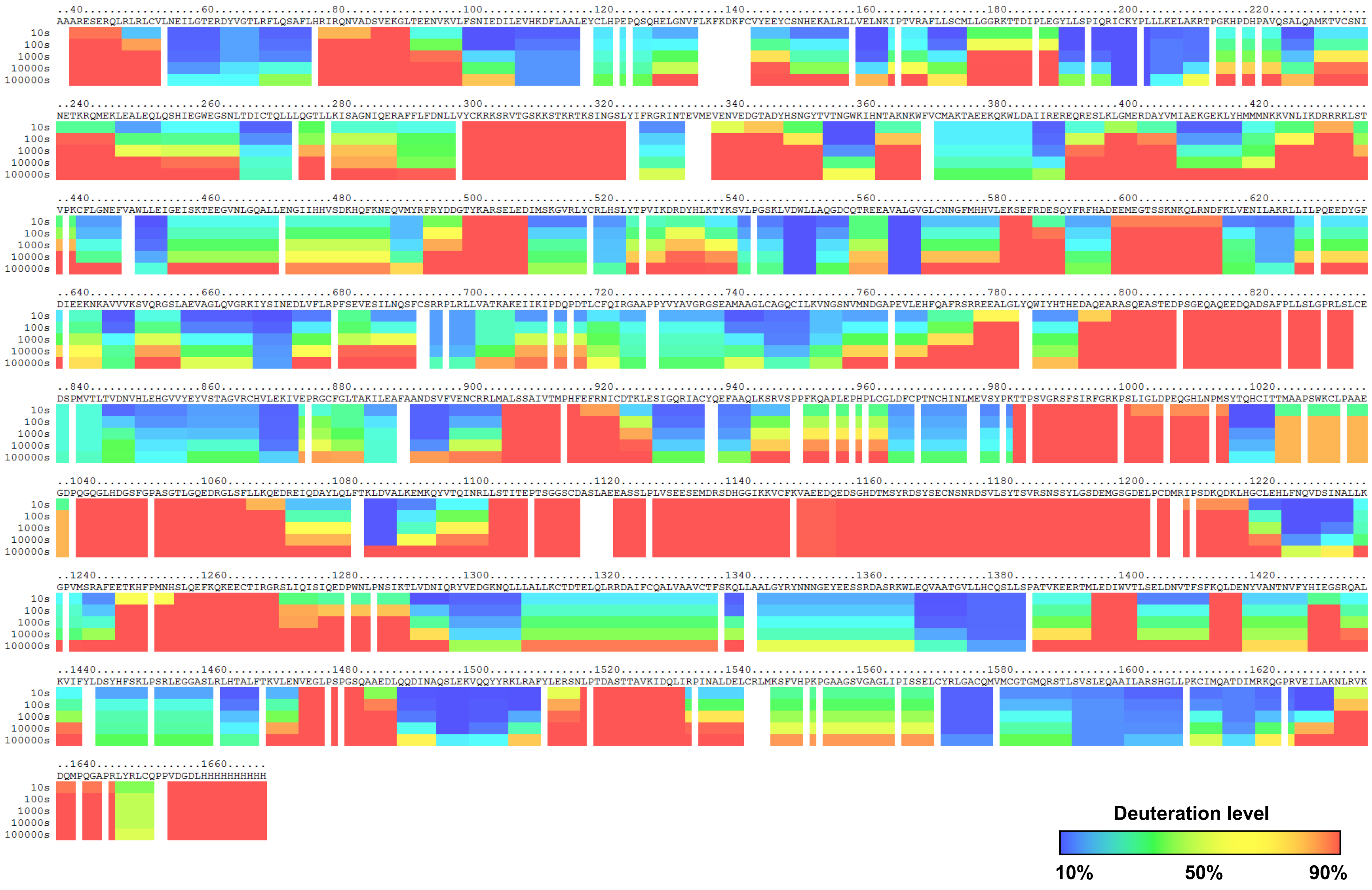

Ribbon Map of P-Rex1 in the presence of liposomes without PIP_3 (% deuteration)

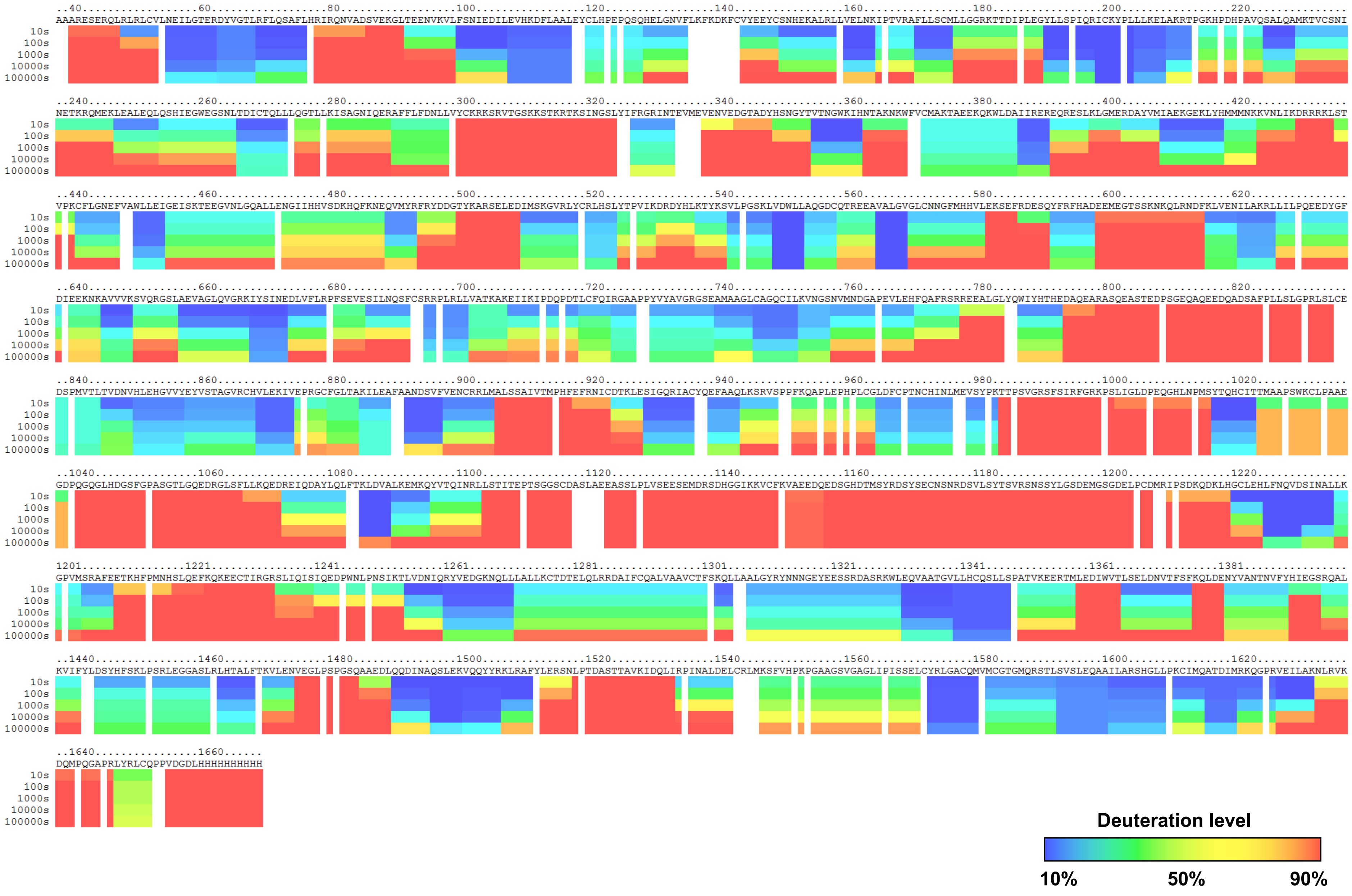

Difference Map: P-Rex1–PIP_3-containing liposomes Minus P-Rex1 (% deuteration)

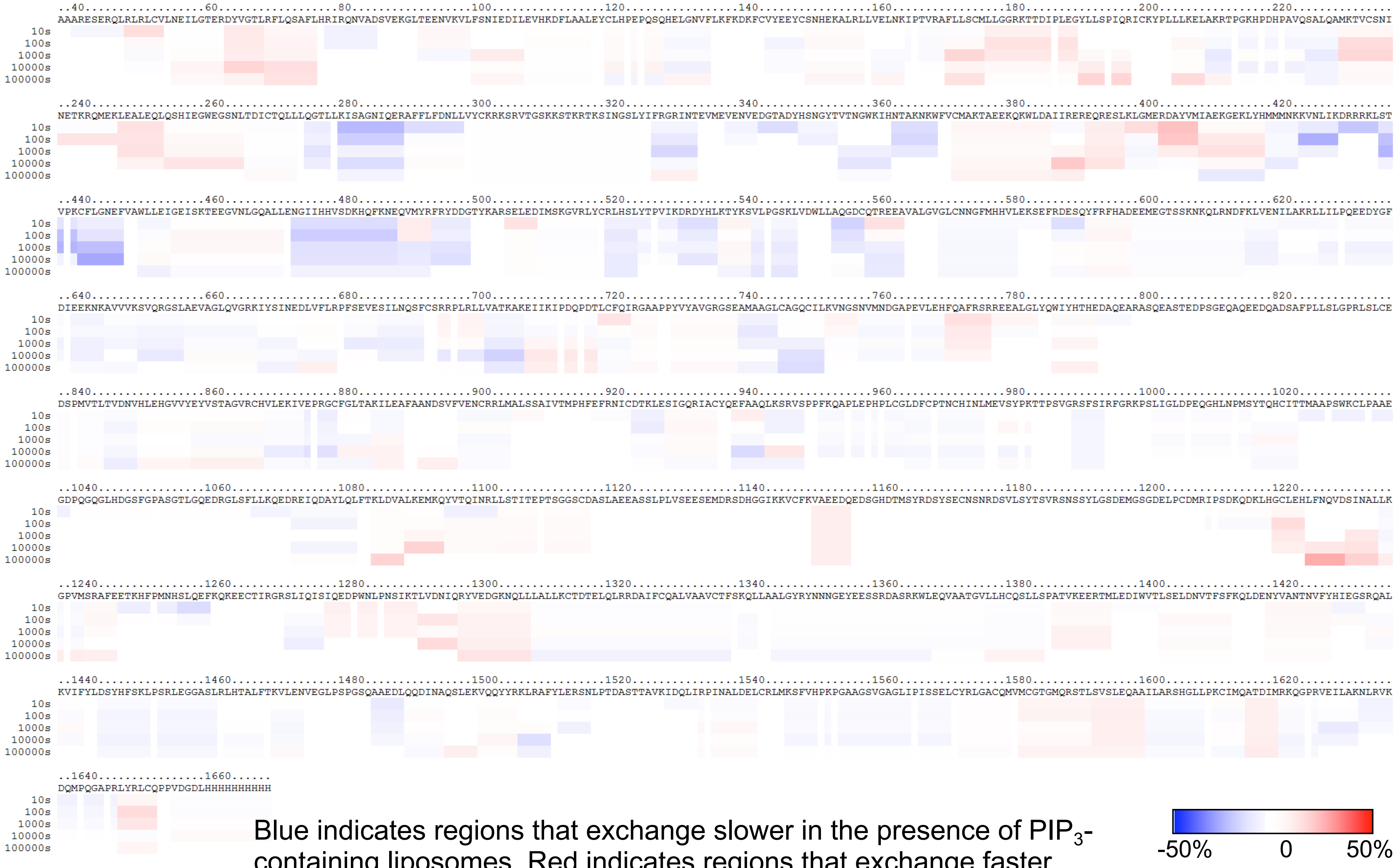

Blue indicates regions that exchange slower in the presence of PIP_3-containing liposomes. Red indicates regions that exchange faster.

Difference Map: P-Rex1–liposomes Minus P-Rex1 (% deuteration)

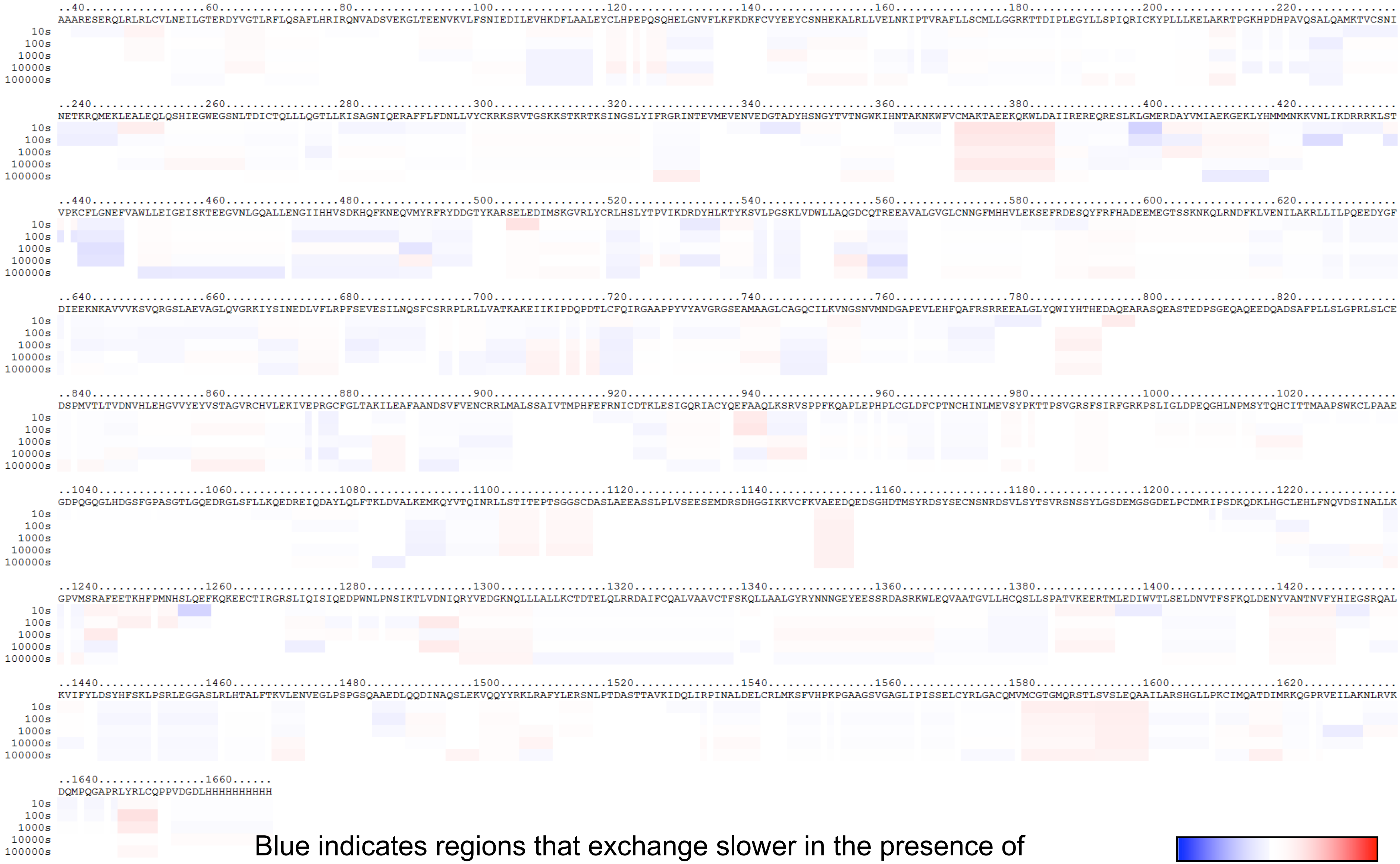

Blue indicates regions that exchange slower in the presence of liposomes without PIP_3. Red indicates regions that exchange faster.

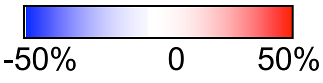

Supplement: Figure 6—source data 1. [file elife-92822-fig6-data1.pdf]
